# Transparent and Flexible Vibration Sensor Based on a Wheel-Shaped Hybrid Thin Membrane

**DOI:** 10.3390/mi12101246

**Published:** 2021-10-14

**Authors:** Siyoung Lee, Eun Kwang Lee, Eunho Lee, Geun Yeol Bae

**Affiliations:** 1 Department of Chemical Engineering, Pohang University of Science and Technology, Pohang 37673, Korea; challenge@postech.ac.kr; 2 MLCC Development Team, Samsung Electro-Mechanics, Suwon 16229, Korea; unisteklee@gmail.com; 3 School of Energy and Chemical Engineering, Center for Dimension-Controllable Organic Frameworks, Ulsan National Institute of Science and Technology (UNIST), Ulsan 44919, Korea; 4 Department of Chemical Engineering, Kumoh National Institute of Technology (KIT), Gumi 39177, Korea; 5 Green and Sustainable Materials R&D Department, Korea Institute of Industrial Technology (KITECH), Cheonan 31056, Korea

**Keywords:** transparency, organic/inorganic hybrid, flexible sensor, vibration sensor

## Abstract

With the advent of human–machine interaction and the Internet of Things, wearable and flexible vibration sensors have been developed to detect human voices and surrounding vibrations transmitted to humans. However, previous wearable vibration sensors have limitations in the sensing performance, such as frequency response, linearity of sensitivity, and esthetics. In this study, a transparent and flexible vibration sensor was developed by incorporating organic/inorganic hybrid materials into ultrathin membranes. The sensor exhibited a linear and high sensitivity (20 mV/g) and a flat frequency response (80–3000 Hz), which are attributed to the wheel-shaped capacitive diaphragm structure fabricated by exploiting the high processability and low stiffness of the organic material SU-8 and the high conductivity of the inorganic material ITO. The sensor also has sufficient esthetics as a wearable device because of the high transparency of SU-8 and ITO. In addition, the temperature of the post-annealing process after ITO sputtering was optimized for the high transparency and conductivity. The fabricated sensor showed significant potential for use in transparent healthcare devices to monitor the vibrations transmitted from hand-held vibration tools and in a skin-attachable vocal sensor.

## 1. Introduction

Vibration sensors have been widely used in industrial applications to detect the operation and status of fans, compressors, chillers, and pumps [1,2,3]. The recent development of human–machine interaction and Internet of Things (IoT) has required vibration sensors to provide more convenience, including to workers, by detecting human voices or surrounding vibrations nearby. For example, the sensors need to detect human voices, abdominal sounds, and heart sounds by measuring the skin vibration [4,5], even in noisy environments [6]. In particular, sensors that monitor long-term and frequent exposure to hand-held vibration tools and whole-body vibrations can help prevent neurological, vascular, and musculoskeletal health problems such as hand–arm vibration syndrome [7,8,9]. For these applications, instead of a bulky vibration sensor, wearable and flexible vibration sensors that are easy to conformally attach to equipment or the human body have been developed [6,10,11,12,13,14].

However, previously reported wearable sensors do not exhibit a high-quality vibration–response performance or sufficient esthetics. Specifically, such sensors have mainly introduced piezoelectric, triboelectric, and piezoresistive mechanisms, and thus, they have achieved a high sensitivity only at a specific resonance frequency and do not have a constant sensitivity over a wide frequency range [13,14]. In addition, the stable detection of external vibrations of various magnitudes and frequencies has been limited because the vibration response over a wide range of vibration amplitudes has been nonlinear and small [10,11]. Furthermore, although a high transparency is a critical characteristic that can be applied to practical wearable sensors from an esthetic and functional perspective [15], previous sensors have not revealed a high transparency owing to the employment of opaque metals as electrode materials [6,12].

In this study, a wearable and flexible vibration sensor with both a high vibration response and high transparency was designed by exploiting a hybrid diaphragm structure with the advantages of both organic and inorganic materials. The device structure consists of a UV-curable polymer SU-8, whose solution-based process and crosslinking property can easily form ultrathin and flexible sensor structures with sophisticated patterns. With a small Young’s modulus, the polymer has less stiffness and thus a higher mechanical response than inorganic materials. In addition, an indium tin oxide (ITO) electrode was introduced to supplement the inherent lack of conductivity of SU-8 as well as to achieve a high transparency and flexibility similar to those of SU-8. The high conductivity of ITO allows the electric signal to be stably output when the vibration sensor detects vibration. In particular, to obtain a high transparency and conductivity of ITO sputtered on a polymer substrate, i.e., SU-8, the annealing temperature during the post-annealing process after ITO sputtering was optimized.

A wearable and flexible sensor of a wheel-shaped capacitive diaphragm structure with an SU-8/ITO membrane was fabricated using a polymer MEMS and a thin film transfer method. The sensor has a flat frequency response within the frequency range of 80–3000 Hz owing to the low structural/material damping effect from the wheel-shaped-patterns and a fully crosslinked SU-8 of the SU-8/ITO membrane. The sensor also shows a linear and high vibrational response of 20 mV/g, which results from the change in capacitance of the large (diameter of 400 µm) and thin (thickness of 2 µm) diaphragm suspended on a diaphragm support (thickness of 2 µm) when the diaphragm moves up and down. The ultrathin structure (≈20 µm) made of a transparent polymer and an ITO electrode not only allows the device to be placed on the skin or curved surface conformally, it also shows a high transparency, causing outstanding practicality and wearability as the device visually harmonizes with the attachment surface. Based on these advantages, the vibration sensor was attached to the back of the user’s hand and was then demonstrated as a healthcare device for detecting the surrounding vibrations by monitoring the vibrations generated from hand-held vibration tools. In addition, the vibration sensor demonstrated the potential use as a skin-attachable and transparent microphone.

## 2. Materials and Methods

### 2.1. Structure and Material Design

**Figure 1:**
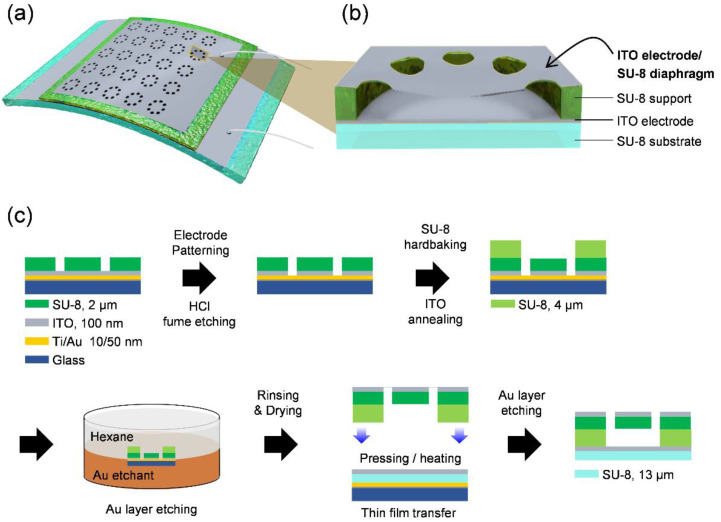
Design and fabrication process of the vibration sensor. (**a**) Schematic design of the transparent and flexible vibration sensor consisting of diaphragm array. (**b**) Schematic of cross-sectional viewed suspended diaphragm of the vibration sensor. (**c**) Overall fabrication process of the vibration sensor.

A flexible vibration sensor consisting of 400 patterned diaphragms with a diameter of 400 µm was designed (Figure 1a) for a clear description of the device structure, where only 25 arrays are schematically represented. The diaphragm, diaphragm support, and substrate consist of SU-8, which is a UV-curable and thermally and chemically stable polymer (Figure 1b). Two transparent ITO electrodes were placed on the diaphragm and substrate, allowing the flexible vibration sensor to achieve a capacitive structure, in which the diaphragms were electrically connected in parallel. The diaphragm array can accumulate changes in the capacitance of each diaphragm, which results in an increase in the output electric signal. The diaphragm moves up and down dynamically by the inertia resisting the vibration when the attached base vibrates, and the capacitance of the diaphragm is changed by the change in distance between the top and bottom ITO electrodes.

### 2.2. Fabrication Process

During the fabrication process of the diaphragm structure (Figure 1c), a Ti/Au film (10/50 nm) was first thermally evaporated onto a cleaned glass carrier wafer as a sacrificial layer. For the top electrode, ITO (100 nm) was sputtered (SRN-120, SORONA). The top electrode was patterned using a shadow mask during the sputtering process. An epoxy resin diaphragm (2 μm) was formed by spin coating a diluted solution (Micro Chem., SU-8 2015) and then patterning the polymer film into a circular array with air holes using a photolithography process (MDA-400M, Midas system, Daejeon, Korea). The ITO electrode exposed to the air hole patterns was removed through HCl fume etching, in which the glass dish containing an HCl solution (35–37%, DAEJUNG) and the sample were placed together in a small chamber for approximately 2 min [16]. After that, the etched sample was rinsed with DI water. The diaphragm/electrode layer was heated for hard baking of the polymer diaphragm by fully crosslinking the epoxy groups of SU-8 and thermal annealing of the ITO electrode to achieve high transparency and conductivity. The heating temperature was optimized within the range of 25 °C (room temperature) to 300 °C. An additional SU-8 layer (4 μm) was spin-coated onto the diaphragm and patterned into a diaphragm support. Then, the sacrificial Au layer was etched using an organic/aqueous biphasic solution of hexane and the Au etchant (Gold etchant, Sigma-Aldrich, St. Louis, MO, USA), which made the interface energy sufficiently low, thereby preserving the diaphragm structure. The Au etchant does not incur a side effect on the ITO electrodes during the etching process of the Au film. The separated diaphragm structure layer (top electrode/diaphragm/diaphragm support) was rinsed with a biphasic solution of hexane and deionized water. The diaphragm structure was scooped using a rigid metal frame with a square hole and dried in air.

Ti/Au (10/50 nm), SU-8 (13 μm), and ITO (100 nm) were sequentially formed onto a glass carrier wafer as a sacrificial layer, substrate, and bottom electrode, respectively. A thin film of SU-8 (20 nm) was spin-coated onto Au as an adhesive layer. The dried diaphragm structure layer was placed onto the bottom electrode with a support layer facing the bottom electrode and pressed while heating at 120 °C. The diaphragm structure and substrate layers were conformally adhered by crosslinking the epoxy groups at the interface between the diaphragm support layer and the adhesive layer [6]. After the sacrificial Au layer was removed with an Au etchant, the top and bottom electrodes were wired using silver paste for electrical characterization. A bio-compatible adhesive (LP-001, ABLE C&C Co., Ltd., Seoul, Korea) was used to attach the vibration sensor on human skin and a curved surface.

### 2.3. Characterization

Top views of the diaphragm structure were obtained using a scanning electron microscope (S-4800, Hitachi, Tokyo, Japan) and an optical microscope (Axioplan, ZEISS, Oberkochen, Germany). The carbon and indium components in the suspended diaphragm were measured through an energy-dispersive X-ray analysis (X-MaxN, HORIBA, Kyoto, Japan). A cross-sectional profile and a 2D contour of the suspended diaphragm were obtained using a 3D profiler (Wyko NT1100, Veeco, Tucson, AZ, USA). The electrical conductivity of the ITO electrode was obtained using four-point probe measurements (PB100, MSTECH, Hwaseong, Korea) and a surface profiler (NanoMap-PS, AEP Technology, Santa Clara, CA, USA). The transmittance of the electrode and polymer layer within the wavelength range of 300–800 nm, including visible light, was measured using a spectrophotometer (V-770, JASCO, Easton, MD, USA). The crystal structure and surface morphology were analyzed using an X-ray diffractometer (D/MAX-2500-PC, Rigaku, Austin, TX, USA) and an atomic force microscope (Multimode-8, Bruker, San Jose, CA, USA). The output electrical signal was measured and stored using oscilloscopes. The real-time fast Fourier transform (FFT) of the output data was carried out using a signal analyzer (SR785, Stanford Research Systems, Sunnyvale, CA, USA). The frequency spectrum of the output signal was obtained using MATLAB (R2016a, MathWorks, Natick, MA, USA).

## 3. Results and Discussion

### 3.1. Optical and Electrical Properties of ITO/SU-8 Layer

The sensor was designed to have ITO/SU-8 layers as the top electrode/diaphragm and bottom electrode/substrate. In general, for ITO to achieve a high transparency and conductivity, a post-annealing process at temperatures ranging from 300 to 700 °C is required after ITO formation [17,18]. However, ITO on a polymer substrate such as PET cannot achieve such a high-temperature post-annealing process and thus has been formed under limited and demanding conditions [19]. To fabricate the proposed sensor, the applicability of the post-annealing process was demonstrated by considering the relatively high glass-transition/melting temperature of SU-8 [20] among various organic materials. After fabricating the ITO/SU-8 bilayer, the annealing process was applied under ambient air pressure from room temperature to 300 °C for 1 h. The heating and cooling rates required to reach the target temperature were set to approximately 4 °C/min.

**Figure 2:**
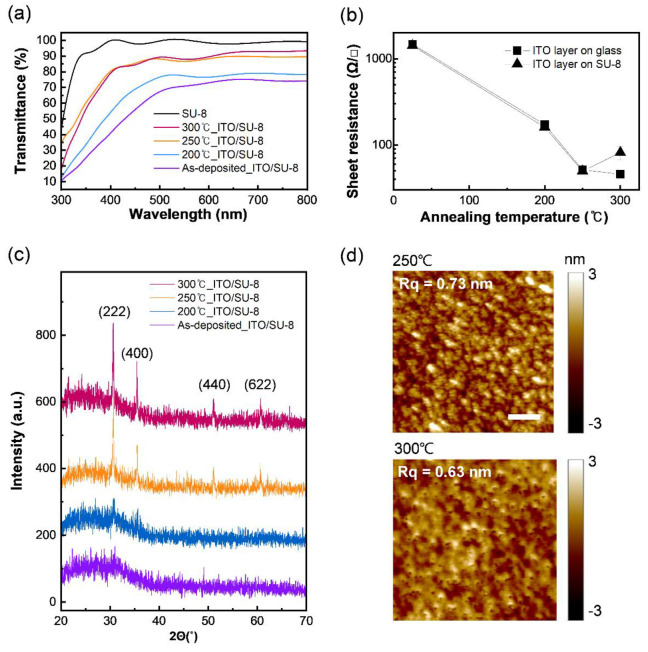
Optical and electrical characteristics of the ITO/SU-8 layers for the vibration sensor. (**a**) Transmittance of the ITO/SU-8 layer within a UV-visible wavelength range (300–800 nm) depending on the temperature of the post-thermal annealing. Black line represents an SU-8 layer without ITO electrode. (**b**) Conductivity of ITO electrode on glass and SU-8 layer depending on thermal annealing. (**c**) X-ray diffraction image of ITO/SU-8 layer depending on the temperature of post-thermal annealing. (**d**) Surface morphology of ITO/SU-8 layer after thermal annealing at 250 °C (Top) and 300 °C (Bottom) (scale bar, 100 nm).

The as-deposited ITO electrode on the SU-8 layer showed ≈70% transmittance at a wavelength of 550 nm (Figure 2a). The comparison between the ITO/SU-8 bilayer (navy line) and the SU-8 monolayer (black line) shows that the transmittance is lowered by the ITO. The transmittance increased significantly as the post-annealing temperature increased up to 250 °C (blue and orange lines) and converged to a similar level at 300 °C (magenta line). According to many previous studies [21,22], the conductivity of ITO increases as the thermal annealing temperature increases because the structure of the ITO films is rearranged and helps the Sn ions become effective dopants [22]. In this work, the sheet resistance of the ITO layer, which was approximately 1473 Ω/□ in the as-deposited sample, decreased to 45.76 Ω/□ at 300 °C (Figure 2b). Considering the thickness (100 nm) of the deposited ITO, the conductivity of the as-deposited ITO was estimated to increase from 67.9 to 581, 1950, and 2190 S/cm as the ITO was thermally annealed at 200 °C, 250 °C, and 300 °C, respectively. The ITO formed on SU-8 showed the same tendency as the ITO on the glass until the thermal annealing temperature reached 250 °C. However, when the thermal annealing temperature reached 300 °C, the conductivity decreased by approximately two-fold in comparison to the case in which the thermal annealing temperature was 250 °C, and a large variation between samples was shown.

The change in transmittance and conductivity by thermal annealing can be explained by the crystalline properties and surface morphology of the ITO electrode on the SU-8 layer. The crystalline properties were analyzed using X-ray diffraction (XRD) data (Figure 2c). The as-deposited ITO layer had no diffraction peaks, indicating the amorphous phase of the film. After the post-annealing process, the diffraction peaks of the (222), (400), (440), and (622) planes could be observed in the ITO layer, which represents a cubic structure (JCPDS: 71-2194) [23]. Sn was successfully integrated into the In^3+^ lattice sites, which was proved by the dominance of the (222) peak over the (400) peak [23], and the fact that the SnO_2_ (26.5°) and SnO (33.2°) peaks were not observed [17]. The (222) peak shifts toward a smaller 2Θ as the annealing temperature increases, which is related to the strain change of the crystal lattice [24] owing to the greater substitution of Sn^4+^ ions into the indium site. The crystallite size (*D*) was determined (Table 1) from the XRD pattern using Scherrer’s formula [17]:

(1) $$D=Κ\frac{\lambda }{\beta cos\theta }$$

where λ denotes X-ray wavelength (λ=1.54060Å), β is the full width at half maximum in radian, θ is the diffraction angle of the peak (222), and Κ represents the shape constant (Κ = 0.94).

During thermal annealing at 200 °C, ITO formed crystals with a D of 34 nm, which significantly improved to 78 nm at 250 °C. When the annealing temperature reached 300 °C, the size of the crystal was found to be slightly small, at approximately 70 nm. As D increases, the grain-boundary scattering of visible light and electrons decreases and the carrier lifetime increases, which affects the transmittance and conductivity of ITO on the SU-8 layer [22]. Thus, the D values of the obtained ITO film can explain why the transparency and conductivity of ITO on SU-8 increased up to the thermal annealing temperature of 250 °C and why these properties did not increase at 300 °C. However, the phenomenon in which the D of ITO sputtered on SU-8 decreases slightly at 300 °C is different from the tendency of D of ITO on glass reported in a previous study [17] in which D continues to increase with increasing temperature. This difference is related to the change in the surface morphology of SU-8. According to a previous study on SU-8 hard baking [20], the epoxy rings and para-substituted benzenes in SU-8 can be decomposed at above 275 °C, and thus, the crosslinking between monomers is broken and structural disorders increase. As a result, it is assumed that the morphology of SU-8 changed and influenced the crystallinity of ITO, which is supported by the surface morphology of thermally annealed ITO on SU-8 measured using an atomic force microscope (Figure 2d); the ITO grains were clearly distinguished in ITO on SU-8 annealed at 250 °C, and the sample annealed at 300 °C had several nanometer-sized pin-holes, which could be related to the decomposition and structural disorders of SU-8. It also supports the result that ITO on SU-8 exhibited higher sheet resistance than on glass, which is not decomposed at the same temperature of 300 °C (Figure 2b). Therefore, to fabricate ITO/SU-8 with high conductivity and transparency, 250 °C was chosen as a suitable post-annealing temperature considering the structural disorder of SU-8, and this annealing method was applied to fabricate the sensor.

### 3.2. Configuration of Flexible Vibration Sensors

**Figure 3:**
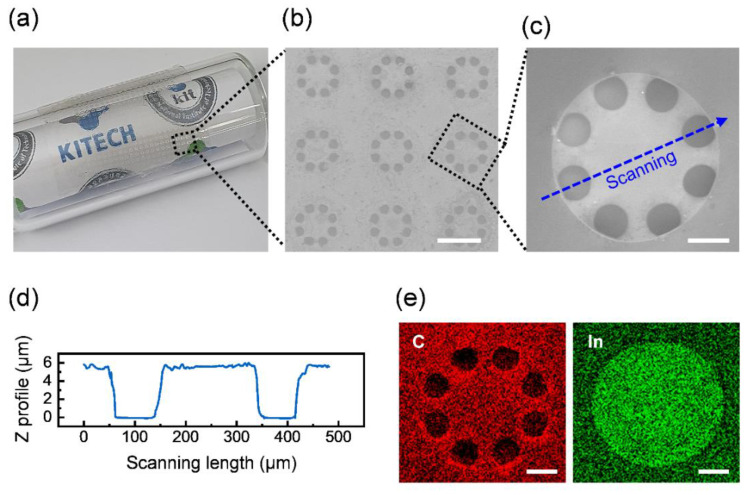
Configuration of the vibration sensor. (**a**) Photographic showing the transparency and flexibility of the vibration sensor. SEM image of (**b**) the diaphragm array (scale bar, 400 nm) and (**c**) a diaphragm structure of the vibration sensor (scale bar, 100 nm). (**d**) Height profile of cross-sectional view for the suspended diaphragm structure. Scanning route of (**d**) is shown in the dashed arrow of (**c**). (**e**) Carbon and indium elemental mapping data obtained using energy-dispersive X-ray mapping analysis for the same diaphragm in the SEM image (**c**) (scale bar, 100 nm).

The fabricated sensor consisting of a thin (≈20 µm) structure with organic SU-8 and inorganic ITO can be conformally attached to a curved glass surface with a bending radius of 7 mm (Figure 3a). The optimized temperature condition of post-thermal annealing for the ITO/SU-8 layer provides a sensor with a high transparency such that the image behind the sensor can clearly be seen (Figure 3a). A diaphragm with a thickness of 2 μm and a diameter of 400 μm were arranged in an array at 700 μm intervals (Figure 3b,c). The diaphragm was suspended on the diaphragm support at a distance of 4 μm from the bottom electrode, and the diaphragm had an initial deflection of less than 100 nm, stably forming an air gap (Figure 3d). The high lateral aspect ratio of the air film (100:1) between the diaphragm and the substrate increases the change in capacitance when the diaphragm is moved by external base vibration. Eight-hole patterns of the wheel-shaped diaphragm with an 80 μm diameter (Figure 3c) are designed to reduce the squeezed air film damping effect that occurs when the diaphragm moves [25] and to lower the stiffness of the diaphragm by connecting the diaphragm edge with thin lines [6]. Carbon and indium elemental mapping images obtained through energy-dispersive X-ray spectroscopy (Figure 3e) show that the structure of the device consists of an organic material, i.e., a polymer, and that the electrode is made of an inorganic material, ITO. Indium can be clearly observed along the pattern of the diaphragm support without a distinction of the hole pattern of the diaphragm owing to the strong influence of the ITO electrode under the suspended diaphragm.

### 3.3. Vibration Response of the Fabricated Sensor

**Figure 4:**
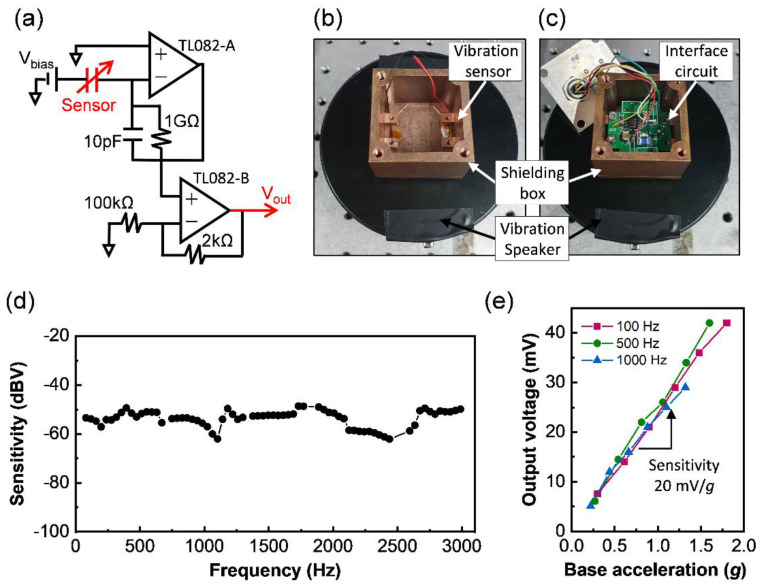
Experimental setup and measurement results for vibrational response of the sensor. (**a**) Interface circuit diagram connected to sensor. Photographic images of (**b**) the device and (**c**) the electric circuit located in a shielding box made of metals on the vibration speaker. (**d**) Frequency response and (**e**) linearity of sensitivity of the fabricated sensor depending on input frequencies.

The fabricated wearable and flexible sensor with a diaphragm structure was wired with an interface circuit composed of a charge and voltage amplifier to measure the vibration response (Figure 4a). The capacitance variation (ΔC) of the diaphragm structure was converted into an electric charge (ΔQ) under a fixed voltage bias (Vo)(ΔQ = ΔC × V0). The bias voltage was set to be sufficiently high to achieve the high sensitivity of the sensor and address the issue of membrane inertia [26]. Then, the accumulated charge was converted into an output voltage signal using the amplifier in the interface circuit. The vibration response was analyzed by measuring the output voltage of the device and comparing it to that of the reference accelerometer (352C33, PCB Piezotronics, Depew, NY, USA), which maintains the same sensitivity (100 mV/g from 10 to 10,000 Hz). An input vibration was generated using an electromagnetic vibration speaker (VBT-001, Newadin Technology, Shenzhen, Guangdong, China) (Figure 4b). The vibration connected with the interface circuit was kept in a metal shielding box to remove electromagnetic effects from the speaker (Figure 4b,c).

The sensitivity of the device was measured over the frequency range of 80–3000 Hz, considering the fundamental voice frequency range (80–250 Hz) and the standard telephony bandwidth (300–3400 Hz). The device exhibited a flat frequency response (Figure 4d), in which the sensitivity was moderately maintained for the target frequency range. This result is attributed to the low material damping of the fully crosslinked polymer material, low structural damping of the hole patterns of a wheel-shaped diaphragm, and higher resonance frequencies than the target input frequencies [6]. The crosslinking of SU-8 restricts the oscillation and conformation of the phenyl rings in the polymer materials and thus enables a low tan δ among various polymers [27]. A 400 µm diameter diaphragm with a wheel-shaped pattern formed using the high machinability of polymers sufficiently ventilates the air beneath the diaphragm into the ambient air [25].

The device shows a high vibrational sensitivity of 20 mV/g (Figure 4e), which is attributed to the low stiffness of the diaphragm owing to the low Young’s modulus and low residual stress of SU-8, and to the hole-patterned and ultrathin wheel-shaped diaphragm structure for large capacitance changes. The vibration sensitivity of the device is comparable to that of commercialized miniaturized accelerometers [28]. The device also shows a linear vibration response for the input vibration range of 0.2–2 g (Figure 4e), which covers the range of neck skin vibrations when humans speak [6] and vibrations generated from hand-held vibration tools [29]. Since the diaphragm has a constant stiffness in the vibration range and is parabolically deflected, the capacitance change of the diaphragm structure is proportional to the moving distance of the diaphragm [6]. The diaphragm shape and the capacitance change depending on diaphragm deflection could be more accurately analyzed by employing numerical experiments based on the mathematical model considering the fringing effect [26].

### 3.4. Application of Fabricated Sensor

**Figure 5:**
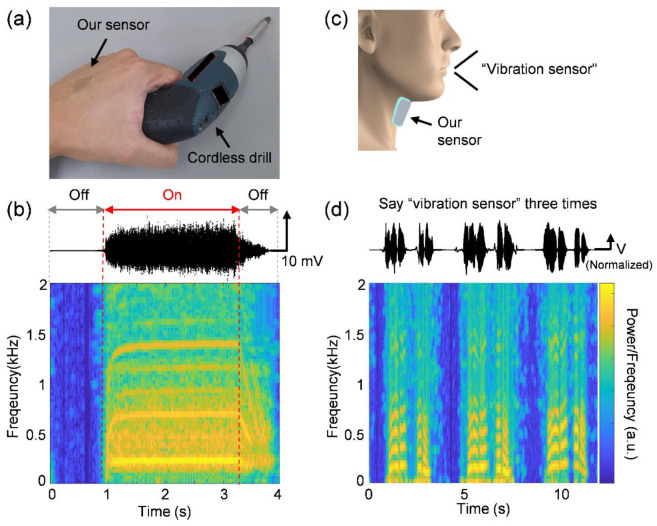
Vibration sensing application of the transparent and flexible vibration sensor. (**a**) Photographic image of the vibration sensor attached to the back of the hand, which grabbed a cordless drill. (**b**) Output waveform and frequency spectrum of the transmitted vibration to the hand measured by the vibration sensor when a cordless drill is turned on and off. (**c**) Schematic of the vibration sensor attached to the human neck skin. (**d**) Output waveform and frequency spectrum of the spoken voice measured by the vibration sensor when a human said “vibration sensor” three times. The output waveforms were filtered by removing electrical noise signals owing to external electromagnetic fields.

With the advantages of high transparency and a vibrational response such as flat frequency response, and linear and high sensitivity, the fabricated sensor demonstrated the potential of wearable and flexible vibration sensors for vibration-related healthcare monitoring and a skin-attachable microphone. First, the sensor was attached to the back of the hand with grabbing a cordless drill, which is a commonly used hand-held vibration tool (Figure 5a). The sensor conformally in contact with human skin provides a high transparency and a harmonious design with human skin. After that, the vibration component transmitted to the hand from the drill was measured using the fabricated sensor. The waveform and frequency spectrum for the output electric signal show that vibration signals of approximately 220 Hz and their harmonics (approximately 440, 660, 880, 1100, and 1320 Hz) were transmitted to the hand during the operation of the drill (Figure 5b). According to the Health and Safety Executive (HSE) of the UK [30], workers should introduce technical and organizational methods to reduce exposure when the hand-arm vibration is over 2.5 m/s^2^. The exposure vibration level should not be over 5.0 m/s^2^ (approximately 0.51 g) to prevent health problems in workers. The measured vibration was less than 0.5 g, considering the amplitude of the output waveform and the sensitivity of the vibration sensor (20 mV/g; Figure 4e). Therefore, the cordless drill is considered to be safe for users, but the workers might be advised to wear vibration-protective gloves for long-term use. As such, by monitoring the vibration transmitted to the hand or body, the fabricated sensor can prevent hand–arm vibration syndrome or whole-body vibration phenomena.

In addition, the device was attached to the neck skin and used to detect the human voice by measuring the neck skin vibration when the human speaks (Figure 5c). The magnitude of the skin vibration acceleration is proportional to the volume of the voice, and the skin vibrates at the same frequency as the voice, and thus, the sensor can detect the voice [6]. The waveform and frequency spectrum of the output electric signal confirmed that the sensor can detect a human voice by measuring the base vibration of the neck skin when the male participant spoke the words “vibration sensor” (Figure 5d). The sensor can also show the stable operation by detecting all of the human voice when the participant repeated the words. With detecting the vibration of the cordless drill and human voice, the transparent and flexible vibration sensor showed the possibility of detecting not only bio-mechanical information generated from the human body but also external mechanical information that can affect the human body.

## 4. Conclusions

In this study, a highly sensitive, transparent, and flexible vibration sensor was developed by employing the advantages of organic and inorganic hybrid materials in a wheel-shaped diaphragm structure. The UV-curable polymer SU-8 with high processability and low stiffness enables the fabricated sensor to achieve a high vibrational response, such as a flat frequency response and high/linear sensitivity. The ITO electrode with high conductivity provides stable vibration detection by helping achieve a stable output of the electrical signal. The high transparency of ITO and SU-8, which is obtained by optimizing the temperature of the post-annealing process, gives the sensor high esthetics. In addition, with an ultrathin structure mainly consisting of a polymer material, the fabricated sensor can be conformally attached to human skin and curved surfaces. The transparent and flexible vibration sensor can monitor the external vibration transmitted from a hand-held vibration tool when the sensor is attached to the hand skin. Moreover, the sensor can perceive the human voice by measuring the vibration acceleration of the human skin. Therefore, the vibration in this work has significant potential for use as a wearable vibration sensor for vibration-related healthcare monitoring and vocal sensing.

## Figures and Tables

**Table 1:** Average crystallite size (D) of the ITO electrode on the SU-8 layer annealed at 200 °C, 250 °C, and 300 °C.

Temperature (°C)	2θ (°) ^1^	β	D (nm)
200	30.78	0.0044	34.42
250	30.66	0.0019	78.21
300	30.64	0.0021	71.69

^1^ Angle of the peak of orientation (222).

## References

[1] Bai L., Zhou L., Jiang X., Pang Q., Ye D. (2019). Vibration in a multistage centrifugal pump under varied conditions. Shock. Vib..

[2] Glowacz A., Glowacz W. (2018). Vibration-based fault diagnosis of commutator motor. Shock. Vib..

[3] Potočnik P., Govekar E. (2017). Semi-supervised vibration-based classification and condition monitoring of compressors. Mech. Syst. Signal Process..

[4] Du X., Allwood G., Webberley K.M., Osseiran A., Marshall B.J. (2018). Bowel sounds identification and migrating motor complex detection with low-cost piezoelectric acoustic sensing device. Sensors.

[5] Bonde A., Pan S., Jia Z., Zhang Y., Noh H.Y., Zhang P. VVRRM: Vehicular vibration-based heart RR-interval monitoring system. Proceedings of the 19th International Workshop on Mobile Computing Systems & Applications.

[6] Lee S., Kim J., Yun I., Bae G.Y., Kim D., Park S., Yi I.M., Moon W., Chung Y., Cho K. (2019). An ultrathin conformable vibration-responsive electronic skin for quantitative vocal recognition. Nat. Commun..

[7] Souissi H., Hamaoui A. (2018). Effect of Human Exposure to Whole-Body Vibration in Transport. Neuroergonomics.

[8] Lindsell C.J., Griffin M.J. (2002). Normative data for vascular and neurological tests of the hand-arm vibration syndrome. Int. Arch. Occup. Environ. Health.

[9] Heaver C., Goonetilleke K., Ferguson H., Shiralkar S. (2011). Hand–arm vibration syndrome: A common occupational hazard in industrialized countries. J. Hand Surg. (Eur. Vol.).

[10] Park J., Lee Y., Hong J., Ha M., Jung Y.-D., Lim H., Kim S.Y., Ko H. (2014). Giant tunneling piezoresistance of composite elastomers with interlocked microdome arrays for ultrasensitive and multimodal electronic skins. ACS Nano.

[11] Kang D., Pikhitsa P.V., Choi Y.W., Lee C., Shin S.S., Piao L., Park B., Suh K.-Y., Kim T.-I., Choi M. (2014). Ultrasensitive mechanical crack-based sensor inspired by the spider sensory system. Nature.

[12] Dinh Le T.-S., An J., Huang Y., Vo Q., Boonruangkan J., Tran T., Kim S.-W., Sun G., Kim Y.-J. (2019). Ultrasensitive anti-interference voice recognition by bio-inspired skin-attachable self-cleaning acoustic sensors. ACS Nano.

[13] Kang S., Cho S., Shanker R., Lee H., Park J., Um D.-S., Lee Y., Ko H. (2018). Transparent and conductive nanomembranes with orthogonal silver nanowire arrays for skin-attachable loudspeakers and microphones. Sci. Adv..

[14] Yang J., Chen J., Su Y., Jing Q., Li Z., Yi F., Wen X., Wang Z., Wang Z.L. (2015). Eardrum-Inspired Active Sensors for Self-Powered Cardiovascular System Characterization and Throat-Attached Anti-Interference Voice Recognition. Adv. Mater..

[15] Bae G.Y., Pak S.W., Kim D., Lee G., Kim D.H., Chung Y., Cho K. (2016). Linearly and highly pressure-sensitive electronic skin based on a bioinspired hierarchical structural array. Adv. Mater..

[16] Oh S., Jung H., Kim Y.-H., Kim M., Yoo E., Choi Y.J., Yoon T.-S., Lee H.H. (2013). Characterization of ITO etching by spontaneously evaporated fume of hydrogen chloride. Microelectron. Eng..

[17] Ali A.H., Hassan Z., Shuhaimi A. (2018). Enhancement of optical transmittance and electrical resistivity of post-annealed ITO thin films RF sputtered on Si. Appl. Surf. Sci..

[18] Seong S., Jung Y.C., Lee T., Park I.-S., Ahn J. (2018). Enhanced uniformity in electrical and optical properties of ITO thin films using a wide thermal annealing system. Mater. Sci. Semicond. Process..

[19] Khachatryan H., Kim D.-J., Kim M., Kim H.-K. (2018). Roll-to-Roll fabrication of ITO thin film for flexible optoelectronics applications: The role of post-annealing. Mater. Sci. Semicond. Process..

[20] Gunde M.K., Hauptman N., Maček M., Kunaver M. (2009). The influence of hard-baking temperature applied for SU8 sensor layer on the sensitivity of capacitive chemical sensor. Appl. Phys. A.

[21] Pokaipisit A., Horprathum M., Limsuwan P. (2008). Vacuum and air annealing effects on properties of indium tin oxide films prepared by ion-assisted electron beam evaporation. Jpn. J. Appl. Phys..

[22] Chan S.-H., Li M.-C., Wei H.-S., Chen S.-H., Kuo C.-C. (2015). The Effect of annealing on nanothick indium tin oxide transparent conductive films for touch sensors. J. Nanomater..

[23] Thirumoorthi M., Thomas Joseph Prakash J. (2016). Structure, optical and electrical properties of indium tin oxide ultra thin films prepared by jet nebulizer spray pyrolysis technique. J. Asian Ceram. Soc..

[24] González G.B., Cohen J.B., Hwang J.-H., Mason T.O., Hodges J.P., Jorgensen J.D. (2001). Neutron diffraction study on the defect structure of indium–tin–oxide. J. Appl. Phys..

[25] Bao M., Yang H. (2007). Squeeze film air damping in MEMS. Sens. Actuator A Phys..

[26] Versaci M., Jannelli A., Morabito F.C., Angiulli G. (2021). A Semi-Linear Elliptic Model for a Circular Membrane MEMS Device Considering the Effect of the Fringing Field. Sensors.

[27] Schmid S., Hierold C. (2008). Damping mechanisms of single-clamped and prestressed double-clamped resonant polymer microbeams. J. Appl. Phys..

[28] Miniature Single Axis Accelerometers of PCB Piezotronics. https://www.pcb.com/sensors-for-test-measurement/accelerometers/miniature-piezoelectric/single-axis.

[29] McGeoch K.L., Lawson I.J., Burke F., Proud G., Miles J. (2005). Diagnostic criteria and staging of hand-arm vibration syndrome in the United Kingdom. Ind. Health.

[30] Health and Safety Executive Hand–Arm Vibration—The Control of Vibration at Work Regulations 2005. www.hse.gov.uk/vibration/hav/regulations.htm.

